# Combining Contrast-Enhanced Mammography and Radioactive-Free Magnetic Seed Localization of Non-palpable Breast Tumors: A Feasibility Study

**DOI:** 10.7150/jca.98597

**Published:** 2024-10-14

**Authors:** Jarn E.M. Theunissen, Els R.M. van Haaren, Caroline N.A. Frotscher, Rachelle R.M. Körver-Steeman, Alfred Janssen, Yvonne L.J. Vissers, James van Bastelaar, Anja Valentijn-Morsing, Lee Bouwman, Marc B.I. Lobbes

**Affiliations:** 1 Zuyderland Medical Center, Department of Medical Imaging, Sittard-Geleen, the Netherlands.; 2 Zuyderland Medical Center, Department of Surgery, Sittard-Geleen, the Netherlands.

**Keywords:** Contrast Enhanced mammography (CEM), Contrast Enhanced Spectral mammography (CESM), Magnetic seed localization (MSL), Breast cancer, Neoadjuvant therapy

## Abstract

**Background:** Magnetic seed localization is a novel and reliable technique for perioperative localization of non-palpable breast cancers. However, due to susceptibility artifacts, magnetic seeds cannot be *in situ* during response monitoring of neoadjuvant chemotherapy with MRI. Contrast-enhanced mammography (CEM) could provide an alternative modality for response monitoring while magnetic seeds are *in situ*. This feasibility study aimed to investigate whether implanted magnetic seeds cause imaging artifacts in CEM examinations.

**Methods:** A phantom experiment and patient studies were conducted to assess the presence of imaging artifacts caused by magnetic seeds on CEM. Chicken breast filet phantoms containing magnetic seeds were imaged using CEM and MRI. Next, twenty women with non-palpable breast tumors scheduled for breast-conserving surgery were included and received a magnetic marker seed preoperatively. Immediately after seed implantation, postprocedural images were taken using the CEM mode on our mammography units. All images were assessed by two experienced breast radiologists for the presence of artifacts. Descriptive statistics were used to present the study results.

**Results:** The phantom experiment revealed no imaging artifacts on CEM, whereas significant artifacts were present on MRI. This allowed us to continue with the patient studies, in which no imaging artifacts associated with magnetic seeds were observed at all. Surgical outcomes demonstrated successful retrieval of all magnetic seeds and negative surgical margins in 19 out of 20 cases.

**Conclusion:** To the best of our knowledge, this is the first study demonstrating that the combination of CEM and magnetic seeds is feasible and does not cause any significant imaging artifacts

## Introduction

The use of neoadjuvant chemotherapy (NAC) in breast cancer treatment has expanded in the last decade 1. Treating breast cancer patients with NAC has several advantages: (1) It increases the rate of breast-conserving therapy due to tumor size reduction 2, 3, (2) NAC serves as an *in vivo* evaluation of treatment response, allowing for treatment alteration when needed 4, 5, and (3) pathological complete response (pCR) after NAC serves as a prognostic factor for survival 6, 7.

However, tumors that have responded well to therapy and perhaps even achieved pCR are more challenging to localize after NAC. To prevent losing the site of the tumor bed, localization markers are often placed within the tumor at the beginning of NAC treatment, when the tumor is still visible with imaging. One of the techniques used for this purpose is radioactive seed localization (RSL) placement using the isotope I-125. RSL is steadily gaining in popularity compared to wire-guided localization for the preoperative localization of non-palpable breast lesions and has become the standard technique in several countries 8. Despite its increasing implementation, RSL also has several disadvantages, including radiation exposure, signal deterioration over time, and rigorous regulations concerning the management and disposal of radioactive material 9. Fortunately, several alternative non-radioactive markers have been developed, including radar reflector-based localization, radiofrequency identification tags, and magnetic seed localization (MSL).

MSL placement before NAC has been proven to be safe and effective for tumor localization 10. Similar to RSL, MSL can often be performed using ultrasound or stereotactic guidance before NAC. MSL can be *in situ* for longer periods of time with the added advantage of no signal deterioration over time 9. The main disadvantage is that both magnetic and paramagnetic marker seeds create substantial susceptibility artifacts on MRI, up to 4-6 cm 9, 11, thus hampering accurate MR imaging assessment of the lesion of interest. This is an important dilemma since MRI is regarded as the imaging reference standard for response monitoring during NAC 12, 13. If the use of MSL before treatment with NAC is to be considered, we need an alternative imaging modality for response monitoring.

**Figure 1:**
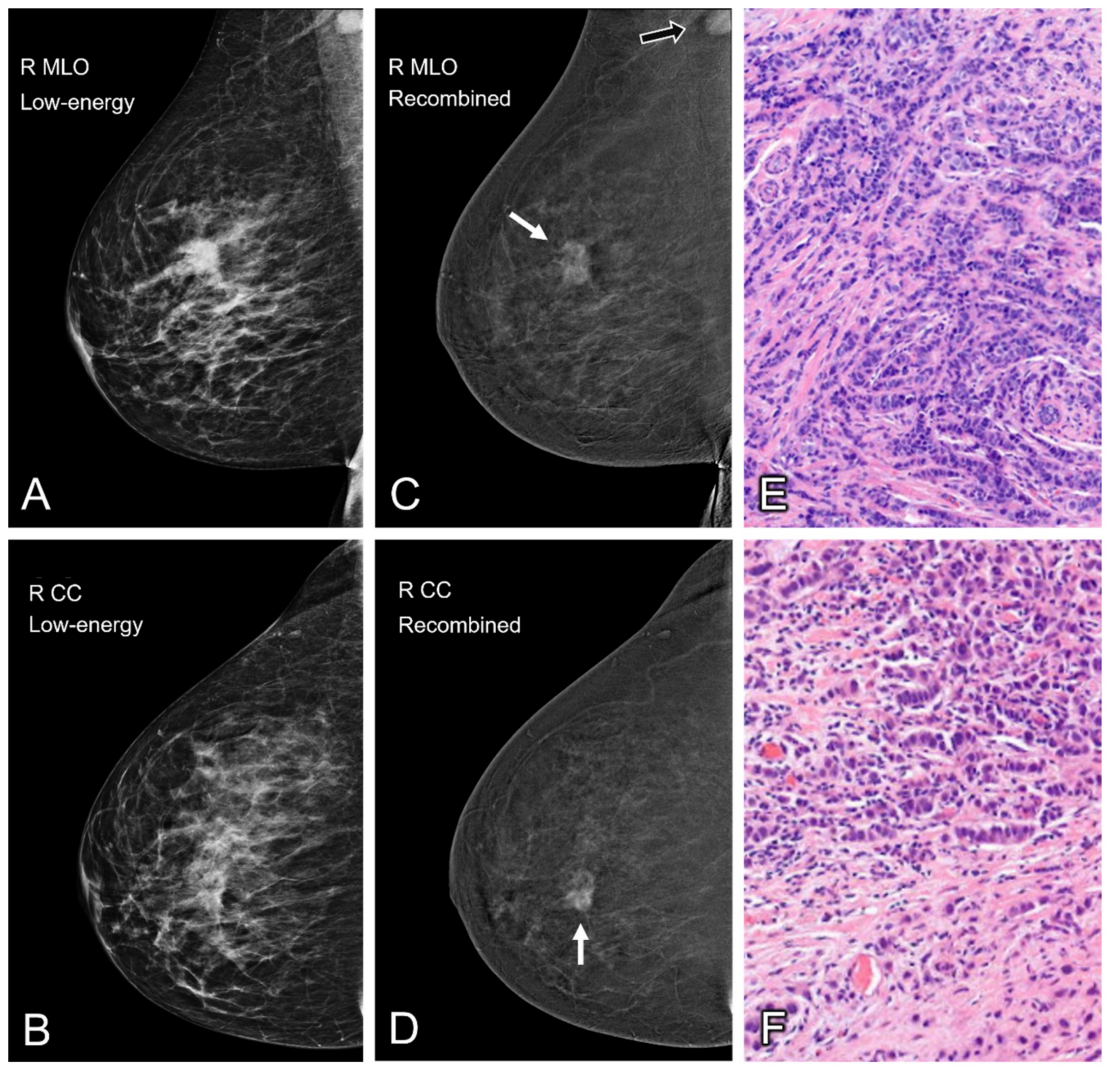
Contrast enhanced mammography of the right breast in both the mediolateral oblique (MLO) and craniocaudal (CC) views. Low-energy images are shown on the left (A and B). The recombined images are displayed in the middle collum (C and D) and show a heterogenous enhancing breast lesion of approximately 2.3 cm (white arrow). A second enhancing lesion can be seen in the right axilla (black arrow). Subsequent histopathological analysis confirmed the presence of invasive carcinoma NST of the breast (E) with axillary metastases (F).

Contrast-enhanced mammography (CEM) has emerged as a promising alternative for this indication. In CEM, a dual-energy mammography is performed two minutes after the intravenous administration of an iodinated contrast agent. Two sets of images are presented to the radiologist of both breasts in at least two views: (1) a low-energy image (which is similar to full-field digital mammography 14) and (2) a recombined image, in which areas of contrast enhancement can be observed (Figure 1). Multiple studies have shown that CEM has a higher diagnostic accuracy than full-field digital mammography 15, even matching the diagnostic accuracy of breast MRI 16. Prior studies have also suggested that CEM might be a suitable alternative to MRI in response monitoring during NAC 17. Since CEM does not use magnetic fields in its image acquisition, no image distortions should occur when CEM and MSL are combined.

This feasibility study aimed to investigate whether implanted magnetic seeds cause imaging artifacts in CEM examinations. First, we tested if image distortions were observed on chicken breast filet phantoms that contained a magnetic seed and that were imaged with CEM and MRI. Second, we tested whether image distortions were observed on CEM in patients who were planned to undergo primary breast-conserving surgery for non-palpable breast lesions, hypothesizing that magnetic seeds do not induce artifacts on CEM images.

## Materials and Methods

### Phantom experiment

Using chicken breast filet to mimic breast tissue, we initially performed a phantom experiment to study our hypothesis *ex vivo*. In this experiment, a magnetic seed (Pintuition Seed®, Sirius Medical B.V., Eindhoven, The Netherlands) was placed in the chicken filet and subsequently imaged using CEM and MRI.

For CEM, both a low-energy and recombined image were acquired of the chicken breast. For MRI, we used a clinical imaging protocol, which consists of transverse T2-weighted imaging and high resolution isotropic T1-weighted imaging. The MRI sequence parameters are summarized in Table 1.

### Patient studies

We included women who were diagnosed with non-palpable invasive breast cancer or ductal carcinoma *in situ* (DCIS) and who met the requirements for primary breast-conserving surgical treatment.

Participants needed to be over 18 years old and not pregnant. There were no further exclusion criteria. This observational feasibility study was approved by the local ethics committee (METC-Z decision number 20220116). Written informed consent was obtained for all study participants. The study was registered at ClinicalTrials.gov (NCT06049446).

Participants underwent preoperative seed localization with the same magnetic seed (Pintuition Seed®, Sirius Medical B.V., Eindhoven, The Netherlands). The seed measures 5.20x1.65 mm and was implanted using a 14 Gauge needle 18. The implementation procedure was performed under local anesthetics and with the help of either ultrasound or stereotactic image guidance. The choice of imaging guidance was determined by the radiologist performing the procedure. Both radiologists performing the procedure were dedicated breast radiologists with clinical experiences of 15 and 12 years.

Post-procedural imaging was performed to confirm the correct positioning of the magnetic seed, using the CEM-mode setting on the mammography unit, resulting in a set of low-energy and recombined images. Since we only wanted to test whether the presence of the magnetic seed would interfere with the image post-processing needed to acquire the recombined CEM image, we decided not to administer an iodinated contrast agent for the post-procedural imaging.

### Imaging analyses

All acquired low-energy and recombined images were centrally assessed by two breast radiologists (M.B.I.L. and C.N.A.F., with breast imaging experience of 15 and 12 years, respectively). For the phantom experiment, the radiologists needed to determine whether or not imaging artifacts were present. For the patient images, radiologists again needed to score ('yes' or 'no') whether imaging artifacts on CEM were present, defining 'imaging artifacts' as image distortions that would have interfered with an accurate (diagnostic) evaluation of the image.

### Surgery

The surgical treatment plan was unchanged by the presence of a magnetic seed, and all surgical procedures were performed by dedicated oncological breast surgeons, with experiences ranging from 13 to 33 years, covering both RSL and MSL procedures. Tumor localization during surgery was performed with a localization system capable of displaying both distance and angle to the magnetic seed (Sirius Pintuition®, Sirius Medical B.V., Eindhoven, The Netherlands). Beyond this change in localization technique, the primary surgical intervention was conducted in adherence to established clinical practice. As surgical outcome parameters, we collected data on surgical margin involvement (positive/negative), tumor diameter (mm), final diagnosis, invasive breast cancer subtypes, and successful seed removal (successful/not successful).

### Statistical analysis

Descriptive statistics were used for both primary and secondary study outcomes. All statistical analyses were performed using IBM SPSS Statistics for Windows, version 27.0 (IBM Corp., Armonk, N.Y., USA).

## Results

### Phantom experiment

**Figure 2:**
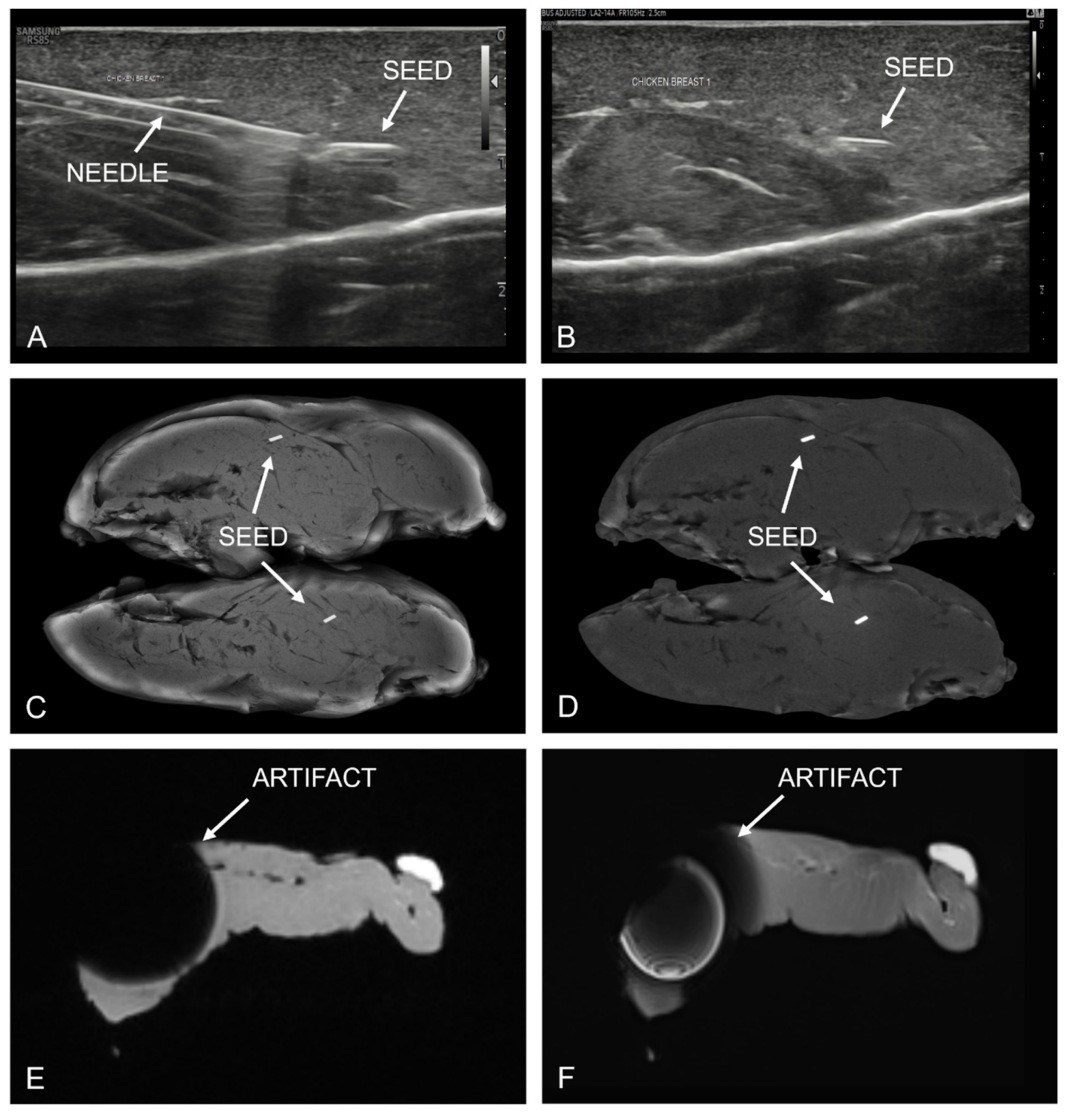
Chicken breast phantom experiment. Top row (images A and B) show placement of the magnetic seed under sonographic guidance, demonstrating good visibility of both the insertion needle and the magnetic seed itself. Middle row shows contrast-enhanced mammography images, consisting of the low-energy (C) and recombined (D) image. In both CEM images, no artifacts were visible due to the presence of a magnetic marker seed. In contrast, the MR images (bottom row) show large susceptibility artifacts on both the T2-weighted TSE sequence (E) and the T1-weighted DIXON sequence (F).

In the phantom experiment, no imaging artifacts were observed at the site of the magnetic seed using CEM. However, substantial imaging (susceptibility) artifacts were observed by both radiologists when using MRI (Figure 2).

### Patient studies

Twenty patients diagnosed with non-palpable breast tumors were included in this study. The mean age was 61 years (range 42-78 years). One lesion was included per patient, this lesion was subsequently localized for surgery using a magnetic seed. No (serious) adverse events were reported during the localization procedure. The implementation procedure was performed with ultrasound image guidance in 15 cases (75%) and stereotactic image guidance in five cases (25%). Seed deployment was successful in all cases.

### Imaging analysis

Low-energy and recombined images were obtained in both the craniocaudal and mediolateral oblique directions of the affected breast only, resulting in four total images per patient with an implanted magnetic seed. In 18 cases, post-procedural imaging was performed on a 3Dimensions™ mammography system (Hologic, Marlborough, MA, USA) using a version of I-View™ 2.0 software with an improved post-processing algorithm for background inhomogeneity removal (pending release). This device was unavailable during two localization procedures due to periodic technical maintenance. Instead, post-procedural imaging for these two cases was performed on a Senographe Pristina™ mammography system (GE Healthcare, Chicago, IL, USA).

**Figure 3:**
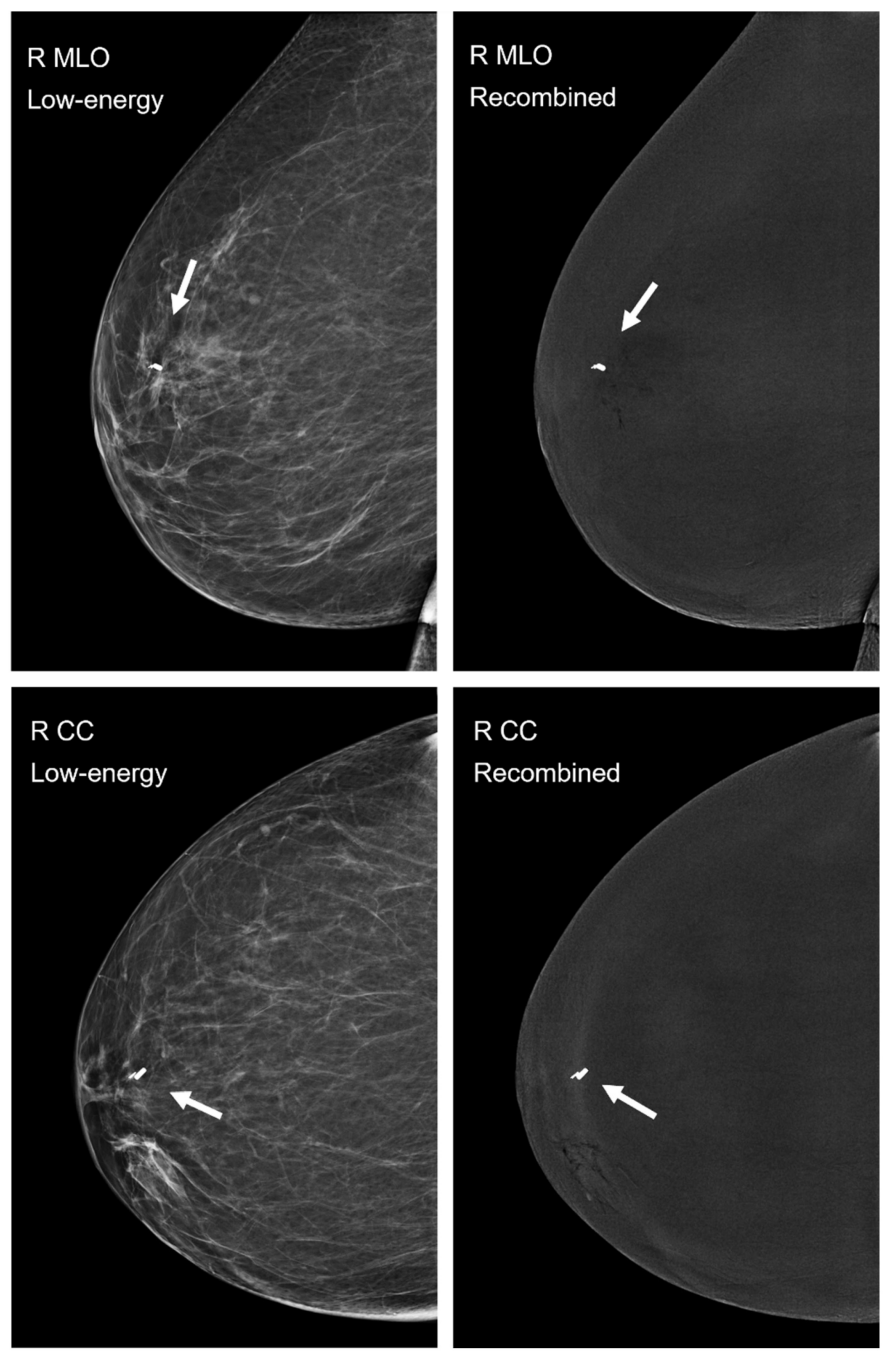
Postprocedural mammography in CEM-mode, low-energy images are displayed on the left, recombined images on the right. Mediolateral oblique (MLO) views are shown in the top row and craniocaudal (CC) views in the bottom row. The magnetic seed is visible in all four images of the right breast (arrow), a smaller localization clip was already *in situ*. None of the examined 40 low-energy or 40 recombined images showed imaging artifacts caused by the implanted magnetic seed.

All 80 images, 40 low-energy and 40 recombined images in both craniocaudal and mediolateral oblique views, were independently assessed by the two experienced breast radiologists. No artifacts associated with the use of the magnetic seeds were identified on any of the images (Figure 3).

### Surgical outcomes

All surgical study results are summarized in Table 2. Intraoperative localization and retrieval of the magnetic seeds were successful in all breast-conserving surgeries (20/20, 100%). The median tumor size was 8 mm. Invasive carcinoma of no special type (NST) was the most predominant diagnosis (14/20 cases, 70%), followed by ductal carcinoma *in situ* (4/20 cases, 20%), and invasive lobular carcinoma (2/20 cases, 10%). Among invasive carcinomas, 20% were classified as grade 1, 55% as grade 2, and 25% as grade 3. Regarding the receptor status, 94% of invasive carcinomas were ER-positive, 75% were PR-positive, and 19% were HER2-positive. Pathological examination revealed negative surgical margins in 19 cases (95%).

In the singular instance where surgical margins were positive for tumor cells, it concerned a DCIS grade 1 with a maximal diameter of 1.0 cm. The surgical margin was deemed more than focally involved at the cranial resection site (>4 mm). Given that the resected cranial margin was located directly underneath the skin, reoperation for additional margin was deemed infeasible. No adjuvant radiotherapy was performed.

## Discussion

In this study, the feasibility of using magnetic seed localization (MSL) in combination with contrast-enhanced mammography (CEM) was investigated. Both a phantom experiment and patient studies were conducted to assess the presence of imaging artifacts caused by magnetic seeds on CEM. In the phantom experiment, no imaging artifacts were found with CEM, whereas substantial imaging artifacts were present with MRI. Likewise, in 20 patients with non-palpable breast tumors, two experienced breast radiologists found no imaging artifacts associated with the implanted magnetic seeds on any of the CEM images (40 low-energy and 40 recombined images). Implementation of the magnetic seed was successful in all 20 cases. Surgical outcomes show successful retrieval of the magnetic seeds in all cases and negative surgical margins in all but one case (95%).

MSL has several advantages over radioactive seed localization (RSL), including sustained signal integrity, absence of radiation exposure, and no extensive administrative regulations. A prospective study of 1,123 magnetic seed placements in non-palpable breast lesions among 1,084 patients demonstrated successful detectability and retrieval of all magnetic seeds. Merely 2.5% of seeds were dislocated, amounting to a correct placement of 97.5%. These results affirm that MSL is an effective and reliable method of preoperative localization 19.

Currently, two magnetic and one paramagnetic commercially available wire-free seed localization systems exist 9: Sirius Pintuition® (Sirius Medical B.V., Eindhoven, The Netherlands), the system employed in this study, alongside MOLLI® (MOLLI Surgical, Toronto, Canada), and Magseed® (Endomag, Cambridge, UK). Magnetic seeds are permanently magnetic whereas paramagnetic seeds are activated by an external magnetic field. Consequently, both types produce significant susceptibility artifacts on MRI 9, 11. Therefore, implementing MSL before neoadjuvant chemotherapy (NAC) hampers accurate response monitoring with MRI, the current imaging reference standard for this indication 12, 13.

The incompatibility of MSL and MRI in response monitoring can be mitigated in two ways. In the first possibility, a radiopaque or ultrasound-visible clip can be placed at the tumor site before NAC, enabling response monitoring with MRI. This clip will subsequently be targeted with MSL shortly before surgery. Mariscal Martínez *et al.* and Reitsamer *et al.* successfully employed this technique to surgically resect targeted metastatic axillary lymph nodes after NAC in cohorts of 30 and 40 patients, respectively 20, 21. However, no studies have currently been published analyzing MSL use after NAC to localize previously clipped breast tumors. This can sometimes be challenging due to poor sonographic visibility of the small clip or in residual breast tumors that responded well to NAC, which occurs more frequently as treatment regimes continue to improve.

A second approach would be to implement MSL before NAC and conduct response monitoring with CEM. Several studies have compared the ability of CEM and MRI to assess response to NAC. Iotti *et al.* 22 conducted a prospective study comparing CEM and MRI in 46 women who underwent both examinations before, during, and after CEM. CEM was superior to MRI in predicting pCR. Sensitivity and specificity for pCR were 100% and 84% for CEM, compared to 87% and 60% for MRI. Barra *et al.* 23 found similar results in a study of 33 patients, with sensitivity and specificity to detect pCR being 88% and 76% for CEM and 75% and 92% for MRI. Patel *et al.* 24 studied 65 patients who underwent both CEM and MRI pre- and post-neoadjuvant systemic therapy and showed similar results. Sensitivity and specificity for pCR were 95% and 66.7% for CEM and 95% and 68.9 % for MRI, respectively. Two other retrospective studies focusing solely on the performance of CEM observed a sensitivity of 86-100% and specificity of 71-83% for pCR 25, 26. Nevertheless, no clinical trials investigating the use of CEM and MSL in response monitoring of women treated with NAC have been conducted. Despite these relatively small number of studies and patients, the results are promising and consistent, suggesting that CEM may be an attractive alternative to breast MRI in response monitoring 27.

Currently, only one study has been published investigating the implementation of MSL in combination with NAC. Malherbe *et al.* 10 retrospectively analyzed a cohort of 21 magnetic seed placements in the breast of 20 patients before or during NAC for palpable breast tumors. The average *in situ* duration of the magnetic seeds was 138 days with all seeds successfully retrieved and no observed migration outside the tumor bed. No response monitoring was performed.

With CEM being a possible alternative to MRI for response monitoring during NAC, the combination of MSL and CEM could provide a novel strategy for women with breast tumors undergoing NAC. To the best of our knowledge, the combination of CEM and MSL has not been evaluated before this feasibility study. The remaining knowledge gaps include the definitive response monitoring capabilities of CEM and prospective data of MSL in patients undergoing NAC. Thus, further research is needed to explore and validate the patient care process combining CEM and MSL in patients undergoing NAC. Our study has proven the feasibility of one part of this process, revealing no significant artifacts when combining MSL with CEM.

Our (feasibility) study has limitations. Firstly, we only included 20 participants for the assessment of the magnetic seed using CEM. As all low-energy and recombined images showed no artifacts associated with magnetic seeds, it is unlikely that increased inclusion numbers would increase the significance of these findings. Secondly, we decided not to administer iodinated contrast. Instead, all post-procedural imaging was conducted in CEM mode on our mammography units without contrast administration. This decision was rationalized by the fact that post-procedural imaging only served to confirm the correct positioning of the magnetic seed and to establish whether magnetic seeds would interfere with the post-processing algorithm for the recombined images. Thus, administrating iodinated contrast was deemed an unnecessary patient burden and therefore unethical. Finally, we understand that restricting our analysis solely to the Sirius Pintuition Seed may raise concerns about generalizability. However, the marker seeds of the three currently wire-free systems share similar dimensions, the Pintuition Seed measuring 5.20x1.65 mm, Magseed 5.0x0.9 mm and MOLLI, 3.2x1.6 mm 18, 19, 28, and all possess a metal density. Therefore, we find it reasonable to assume that if no artifacts are created by the Pintuition Seed, the same applies to the other available magnetic marker seeds.

## Conclusion

In conclusion, the combination of magnetic marker seeds is not causing imaging artifacts on low-energy and recombined images during contrast enhanced mammography. To the best of our knowledge, this study is the first to evaluate the combination of magnetic seed localization and contrast-enhanced mammography.

## Figures and Tables

**Table 1:** Phantom MRI protocol

Vendor	Siemens
Field strength (Tesla)	3.0
T2w sequence	T2 turbo spin echo
In plane resolution (mm)	0.9 x 0.9
Images slice thickness (mm)	1.5 x 1.5
High resolution T1w sequence	T1 3D isotropic voxels, fat suppression
In plane resolution (mm)	0.9 x 0.9
Slice thickness (mm)	0.9

**Table 2:** Overview of study results

Parameter	*n* = 20
Age (median, range)	61 years (42-78)
Implementation procedure (%)	
Ultrasound	15/20 (75%)
Stereotactic	5/20 (25%)
Successful marker deployment (%)	20/20 (100%)
Post-procedural imaging unit used (%)	
Hologic 3Dimensions	18/20 (90%)
GE Healthcare Pristina	2/20 (10%)
Interval insertion-surgery (median, range)	7 days, range 1-9 days
Successful surgical marker retrieval (%)	20/20 (100%)
Tumour diameter (median, range)	8 mm, range 0-110 mm
Negative margins (%)	19/20 (95%)
Final Diagnosis (%)	
Ductal carcinoma *in situ*	4/20 (20%)
Invasive carcinoma NST	14/20 (70%)
Invasive lobular carcinoma	2/20 (10%)
Tumour Grade (n (%))	
Grade I	4/20 (20%)
Grade II	11/20 (55%)
Grade III	5/20 (25%)
Invasive breast cancer subtypes (%)	
ER positive	15/16 (94%)
PR positive	12/16 (75%)
HER2 positive	3/16 (19%)
Image artifacts on CEM (%)	
Not present	20/20 (100%)
Present	0/20 (0%)

Abbreviations; NST: No Special Type; ER: Estrogen Receptor; PR: Progesterone Receptor; HER2: Human Epidermal Growth Factor Receptor 2; CEM: Contrast-Enhanced Mammography.
